# Validation of the manufacturing process used to produce long-acting recombinant factor IX Fc fusion protein

**DOI:** 10.1111/hae.12451

**Published:** 2014-05-08

**Authors:** J McCue, D Osborne, J Dumont, R Peters, B Mei, G F Pierce, K Kobayashi, D Euwart

**Affiliations:** Biogen IdecCambridge, MA, USA

**Keywords:** haemophilia B, human cell line, manufacturing, recombinant, rFIXFc, viral clearance

## Abstract

Recombinant factor IX Fc (rFIXFc) fusion protein is the first of a new class of bioengineered long-acting factors approved for the treatment and prevention of bleeding episodes in haemophilia B. The aim of this work was to describe the manufacturing process for rFIXFc, to assess product quality and to evaluate the capacity of the process to remove impurities and viruses. This manufacturing process utilized a transferable and scalable platform approach established for therapeutic antibody manufacturing and adapted for production of the rFIXFc molecule. rFIXFc was produced using a process free of human- and animal-derived raw materials and a host cell line derived from human embryonic kidney (HEK) 293H cells. The process employed multi-step purification and viral clearance processing, including use of a protein A affinity capture chromatography step, which binds to the Fc portion of the rFIXFc molecule with high affinity and specificity, and a 15 nm pore size virus removal nanofilter. Process validation studies were performed to evaluate identity, purity, activity and safety. The manufacturing process produced rFIXFc with consistent product quality and high purity. Impurity clearance validation studies demonstrated robust and reproducible removal of process-related impurities and adventitious viruses. The rFIXFc manufacturing process produces a highly pure product, free of non-human glycan structures. Validation studies demonstrate that this product is produced with consistent quality and purity. In addition, the scalability and transferability of this process are key attributes to ensure consistent and continuous supply of rFIXFc.

## Introduction

Current haemophilia B management involves replacement therapy with coagulation factor IX (FIX) concentrates, either given episodically (on demand) to treat bleeding episodes or prophylactically to maintain minimum FIX activity and to prevent bleeding 1–4. FIX replacement therapy has been available since the 1970s, initially utilizing plasma-derived FIX concentrates 1,2. Development of recombinant FIX (rFIX) in the 1990s circumvented viral safety concerns associated with plasma-derived concentrates and greatly reduced supply shortages 5 and continues to be important in the treatment of haemophilia B 6.

Due to viral contamination issues with previous plasma-derived FIX products, it is important to characterize the manufacturing process of any new haemophilia therapy and to conduct validation studies that evaluate product quality and demonstrate the ability of the process to remove process-related impurities and viruses. Similar characterization has been done for other rFIX products 7,8. Recombinant FIX Fc Fusion Protein (rFIXFc; Alprolix; Biogen Idec, Cambridge, MA, USA) is the first of a new class of bioengineered long-acting clotting factors. rFIXFc is composed of a single molecule of rFIX covalently fused to the Fc domain of human immunoglobulin G1 (IgG1). The presence of Fc in rFIXFc enables binding to endogenous neonatal Fc receptor (FcRn), a naturally occurring pathway that delays lysosomal degradation of Fc-containing proteins by cycling them back into circulation 9,10. The enhanced pharmacokinetic properties, prolonged half-life, safety and efficacy of rFIXFc have been described in animal models 11 and clinical studies 12,13.

The objective of this work is to describe the rFIXFc manufacturing process and validation studies demonstrating the capacity of this process to produce a highly pure product free from virus.

## Materials and methods

### Manufacturing process: development of the rFIXFc cell line, cell bank creation and cell line confirmation

The coding sequences for human FIX, the Fc region of the human IgG1 (hinge and CH2 and CH3 domains) and PC5 were obtained by a polymerase chain reaction (PCR) from human liver mRNA, human lymphocyte cDNA and human liver mRNA, respectively, and were used to establish the rFIXFc manufacturing cell line 11. Human embryonic kidney (HEK) 293H cells (Life Technologies, Grand Island, NY, USA), preadapted for serum-free suspension culture, were stably transfected with a plasmid containing a dual expression cassette for a FIXFc and Fc chain, a second plasmid expressing Fc alone, and a third plasmid expressing PC5, an enzyme used to ensure complete processing of the FIX propeptide 11,14. Transfected HEK 293H cells were grown in serum-free medium. Clonal cell lines were derived and the optimal cell line selected was based on considerations of rFIXFc monomer productivity, rFIXFc activity [measured by a one-stage clotting assay based on activated partial thromboplastin time (aPTT)], propeptide processing, cell growth properties and stability. The cell lines with the optimal characteristics were then subcloned by limiting dilution and further characterized, and the clonal production cell line was chosen for manufacturing.

The clonal cell line that was selected for manufacturing was expanded to create a research cell bank (RCB). The RCB was expanded to create the master cell bank (MCB), from which a working cell bank (WCB) was derived. The WCB was created by thawing one MCB vial that was serially expanded to obtain sufficient cells for banking. The MCB and WCB were tested for identity, purity and freedom from adventitious agents. The cell banks were stored under liquid nitrogen to ensure long-term stability. The transgene coding sequence, copy numbers and gene integration patterns of the MCB and a cell bank produced from a cell culture that was propagated beyond the end of the manufacturing process [extended end-of-production cell bank (EEPCB)] were compared. The comparison was used to assess and confirm transgene integration and stability of the cell line over the course of the manufacturing process.

### Production of rFIXFc

**Figure 1:**
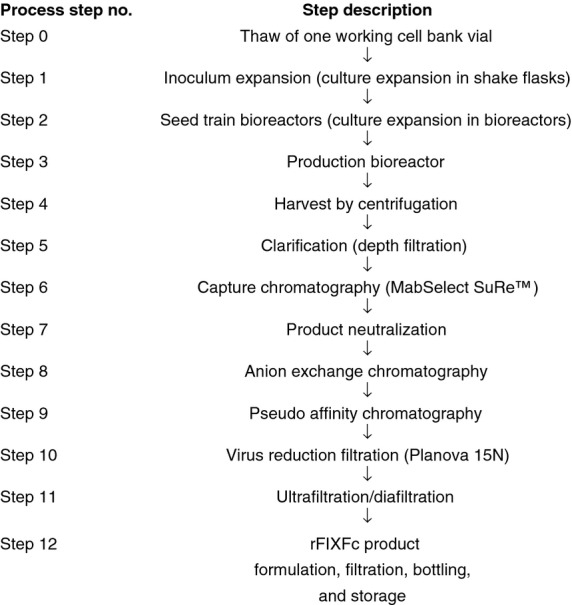
Overview of the recombinant factor IX Fc fusion protein (rFIXFc) manufacturing process.

A WCB vial was used to produce a single batch of rFIXFc in a multi-step manufacturing process outlined in Fig.1 and described below. The inoculum preparation phase includes thawing a WCB vial (Step 0) and expansion of culture in shake flasks (Step 1). Shake flasks were then pooled and used to inoculate the first seed train bioreactor (Seed Train Bioreactor Phase) for further culture expansion (Step 2). The seed train bioreactors were operated in batch mode, with agitation, pressure, temperature, pH and dissolved oxygen controlled during the process. The expanded culture was used to inoculate a large-scale production bioreactor (Step 3), the function of which is to express and produce rFIXFc prior to subsequent harvest and purification operations. The production bioreactor was operated in fed-batch mode, with agitation, pressure, temperature, pH and dissolved oxygen controlled. Harvest of the production bioreactor was initiated at a set time following completion of the final nutrient feed.

Following completion of the cell culture process in the production bioreactor, cells and cellular debris were removed by centrifugation (Step 4) and subsequent depth filtration steps (Step 5) to produce a clarified cell culture harvest containing the rFIXFc product. The product was then initially captured and purified from the clarified cell culture harvest using a protein A affinity capture chromatography step (MabSelect SuRe™; GE Healthcare Bio-Sciences AB, Björkgatan, Uppsala, Sweden) (Step 6), which binds to the Fc portion of the rFIXFc molecule with high affinity and specificity. After elution from the MabSelect SuRe adsorbent and neutralization (Step 7), rFIXFc was further purified using an intermediate anion exchange chromatography step (Step 8) and a polishing anion exchange chromatography step performed in pseudo affinity mode (Step 9). After these three chromatography steps, rFIXFc was passed through a 15 nm pore size virus removal filter (Planova 15N; Asahi Kasei Bioprocesses, Inc., Glenview, IL, USA) (Step 10). The product was concentrated and buffer exchanged using an ultrafiltration step (Step 11) to form the bulk product. The bulk product was filtered into bottles (Step 12) and stored at −70°C.

### Manufacturing process validation studies

Process validation studies were performed to confirm identity, purity, quality and activity of the rFIXFc product. A summary of the analytical tests used in these assessments is shown in Table1. Sodium dodecyl sulphate polyacrylamide gel electrophoresis (SDS PAGE) gels obtained under reducing and non-reducing conditions and stained with colloidal Coomassie were used for validation studies for purity assessment and identity confirmation. The potency [in International Units (IU)] was determined using the one-stage (aPTT) clotting assay against the World Health Organization (WHO) international standard for FIX concentrate, and was used to calculate the specific activity of rFIXFc. The level of activated rFIXFc was monitored for all batches, as activated FIX (FIXa) has been implicated with increased thrombogenic risk 15, and therefore, a sensitive FIXa enzyme-linked immunosorbent assay (ELISA) was developed utilizing an antithrombin III capture step 11. Safety determination was based on testing for the presence of bioburden and endotoxin.

**Table 1:** Analytical tests used to assess identity, purity, activity and safety of rFIXFc

Test	Method description
Polyacrylamide gel electrophoresis (reducing and non-reducing)	Polyacrylamide gel electrophoresis performed in the presence of sodium dodecyl sulphate (SDS PAGE) under both reducing and non-reducing conditions. Gels were stained using Colloidal Coomassie Blue staining
Size exclusion chromatography	Resolution of aggregated forms from the monomeric form of rFIXFc using high performance liquid chromatography (TSK_gel_G3000SW_XL_ column)
Coagulation activity	One-stage aPTT method performed in accordance with Pharmacopeia guidelines (USP<32> and Ph.Eur. 2.7.11) with respect to WHO International Standard for Factor IX concentrate
FcRn binding	FcRn competitive binding measured using an amplified luminescent proximity homogenous assay
Activated rFIXFc	Enzyme-linked immunosorbent assay (ELISA) based on the binding of hATIII to activated factor IX for the detection of the activated form of rFIXFc
Bioburden	Microbial enumeration test performed in accordance with Pharmacopeia guidelines (USP<61> and Ph.Eur. 2.6.12)
Endotoxin	Kinetic turbidimetric method in accordance with Pharmacopeia guidelines (USP<85> and Ph. Eur. 2.6.14)

aPTT, activated partial thromboplastin time; FcRn, neonatal Fc receptor; hATIII, human antithrombin III; rFIXFc, recombinant factor IX Fc fusion protein; SDS PAGE, sodium dodecyl sulphate polyacrylamide gel electrophoresis; USP, United States Pharmacopeia; WHO, World Health Organization.

The purification process was designed to provide a pure product with a high level of viral clearance. To demonstrate the capacity and robustness of the manufacturing process to remove adventitious viruses, the purification process was evaluated for the capacity for removal of enveloped and non-enveloped viruses using four model viruses [xenotropic murine leukaemia virus (X-MLV), mice minute virus (MMV), mammalian orthoreovirus 3 (Reo-3; also known as reovirus serotype 3) and suid herpes virus 1 (SuHV-1; also known as pseudorabies virus)]. These studies were conducted according to the International Conference on Harmonisation Q5A-E Guidelines and the US Food and Drug Administration Points to Consider 16,17 and in accordance with Good Laboratory Practice 18. The virus clearance results were compared to those previously reported for a rFIX product produced in Chinese hamster ovary (CHO) cells 8.

The rFIXFc manufacturing process was also evaluated for the capacity to remove process-related impurities. Process-related impurity clearance validation studies were performed both at the manufacturing scale and in scaled-down spiking studies at the laboratory scale. The clearance validation studies performed at the manufacturing scale consisted of direct measurement of the impurities obtained from the manufacturing process intermediates, in which clearance was calculated from the amount removed during the process step. Scaled-down impurity clearance validation studies (performed at the laboratory scale) consisted of the addition of an impurity to the process intermediate, followed by purification of the spiked intermediate using a scaled-down chromatography step. Scaled-down clearance validation studies were employed for several of the impurities for cases in which the impurity was below detectable levels in the manufacturing process intermediates, and were used to demonstrate the capacity and robustness of the process to provide additional clearance.

## Results

### Cell line safety

MCB and WCB were manufactured in accordance with current good manufacturing practice procedures, with purity, safety and identity test results demonstrating no detectable virus or adventitious agents. Table2 shows all test results for the MCB, WCB and EEPCB. Testing confirmed the cell bank origin and that they are free of bacteria, fungi, mycoplasma and adventitious viruses.

**Table 2:** Cell bank test results for identity, adventitious viruses and endogenous agents

Test/Assay	MCB	WCB	EEPCB
Identity			
Species	Confirmed as human (RAPD)	Confirmed as human (RAPD)	Confirmed as human (isoenzyme analysis)
mRNA sequence (RT-PCR)	rFIXFc sequences according to consensus	Not performed	rFIXFc sequences according to consensus
Microbial tests	Negative	Negative	Negative
Viral tests			
*In vitro* assay for the detection of adventitious virus	None detected	None detected	None detected
Bovine adventitious virus	None detected	Not performed	None detected
Porcine adventitious virus	None detected	Not performed	None detected
*In vivo* assay for the detection of adventitious virus	None detected	Not performed	None detected
PCR screen for human viruses	None detected	Not performed	None detected
TEM (Virus-like particles)	Negative	Not performed	Negative

EEPCB, extended end-of-production cell bank; MCB, master cell bank; PCR, polymerase chain reaction; RAPD, random amplified polymorphic DNA; RT-PCR, reverse transcriptase polymerase chain reaction; TEM, transmission electron microscopy; WCB, working cell bank.

### Manufacturing process validation studies: assessment of product quality and process consistency

The rFIXFc manufacturing process (Fig.1) produced product of consistent purity, quality and activity. All steps in the manufacturing process were successfully validated for consistency based on process performance and product quality data from four batches. The manufacturing process validation study demonstrated the consistency of the rFIXFc process through evaluation of in-process measurements, in-process tests and product quality. Results from four validation batches are shown in Table3.

**Table 3:** Product quality results from four validation batches

		Results			
Product attribute	Test method	LP5-10-FIX-008	LP5-10-FIX-009	LP5-11-FIX-001	LP5-11-FIX-003
Identity	Non-reducing and reducing SDS PAGE; colloidal Coomassie staining	rFIXFc single chain and Fc single chain were identified under reducing conditions. Non-reducing conditions showed one single band of rFIXFc			
Purity	Non-reducing gel electrophoresis (%)	98.3	98.5	98.5	97.6
	Size exclusion chromatography (%)	99.3	99.1	99.0	99.0
Activity	Coagulation activity based on aPTT specific activity (IU/nmol rFIXFc)	7.1	6.9	6.8	6.5
	FcRn binding relative potency^*^ (%)	118	110	104	124
Activated rFIXFc	ELISA (mol%)	0.002	0.003	0.003	0.004
Safety	Bioburden (CFU 10 mL^−1^)	0	0	0	0
	Endotoxin (EU mL^−1^)	<0.20	<0.20	<0.20	<0.20

aPTT, activated partial thromboplastin time; CFU, colony-forming units; ELISA, enzyme-linked immunosorbent assay; EU, endotoxin units; FcRn, neonatal Fc receptor; rFIXFc, recombinant factor IX Fc fusion protein; SDS PAGE, sodium dodecyl sulphate polyacrylamide gel electrophoresis.

One good manufacturing practice (GMP) batch manufactured using the same process, scale and facility has been designated as a reference standard. Relative potency was determined against this reference standard.

**Figure 2:**
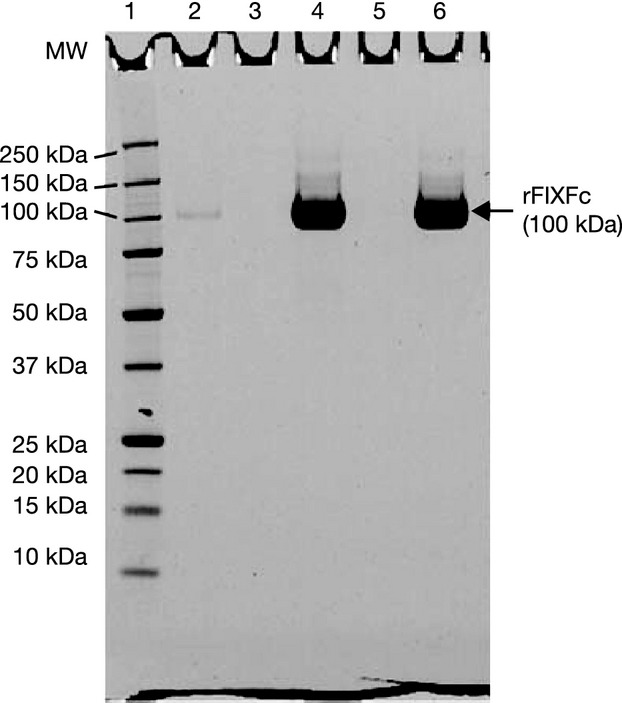
SDS PAGE analysis of rFIXFc from a validation batch (Batch No. LP5-10-FIX-009) for identification and purity determination under non-reducing conditions. Non-reducing SDS PAGE was conducted on a 4–12% polyacrylamide gel in Bis-Tris buffer. Samples were denatured with SDS in the presence of 15 mM N-ethylmaleimide for 15 min at 25°C. The gel was stained with Colloidal Coomassie. Lane numbers: (1) MW markers; (2) rFIXFc assay sensitivity standard, 0.018 μg mass loading; (4) rFIXFc Reference standard, 6 μg mass loading; (6) rFIXFc product (Batch No. LP5-10-FIX-009), 6 μg mass loading; (3, 5) Blank lanes. MW, molecular weight; rFIXFc, recombinant factor IX Fc fusion protein; SDS PAGE, sodium dodecyl sulphate polyacrylamide gel electrophoresis.

**Figure 3:**
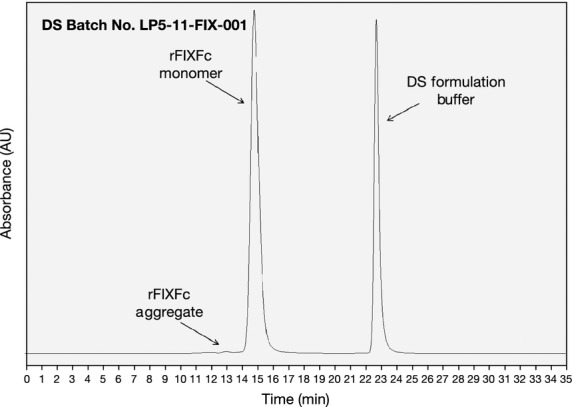
Size exclusion chromatography (SEC) chromatogram of rFIXFc DS Batch No. LP5-11-FIX-001. SEC analysis was conducted on a TSK_gel_G3000SW_XL_ column (Tosoh Bioscience, King of Prussia, PA, USA) using a mobile phase of 0.1 M sodium phosphate/0.1 M sodium chloride pH 6.5. Elution was monitored based on UV absorbance at 214 nm. rFIXFc, recombinant factor IX Fc fusion protein; DS, drug substance.

Overall, results from the analytical testing demonstrated that the manufacturing process consistently produced a highly pure and active rFIXFc product. The results illustrate that the rFIXFc product possesses a high degree of purity, with all batches demonstrating >97% purity, when measured by non-reducing SDS PAGE (Fig.2) and size exclusion chromatography [SEC (Fig.3)]. Results of the SDS PAGE analysis of the rFIXFc product (under non-reducing conditions) illustrate a highly pure product, with a single band present with an observed molecular weight of 100 kDa (Fig.2). As shown in Fig.3 and Table3, SEC was used to confirm that very low levels of aggregates (≤1.0%) were present in all of the batches, further illustrating that a highly pure rFIXFc product was manufactured using the described process.

Table3 also shows that the specific activity of rFIXFc was consistent with validation batches possessing specific activity values of 6.5–7.1 IU nmol^−1^ of rFIXFc, similar to levels reported previously 11. In addition, binding of rFIXFc to FcRn was consistent across the validation batches, with relative binding levels ranging from 104 to 124%. Using a sensitive ELISA for assessment, the level of activated rFIXFc was found to be consistently low (0.002–0.004 mol%) in the validation batches (Table3), which were significantly lower than levels reported for an approved rFIX product 11. In addition, no bioburden was detected in any of the batches, while endotoxin levels were all below detectable levels. Additional analytical characterization was performed with the purified product (data not shown), which further illustrated that product quality was consistent across the validation batches.

### Validation studies: virus- and process-related impurity clearance

The results of virus clearance studies support that the manufacturing process demonstrates robust clearance of viruses possessing different physical and chemical properties.

The capture affinity chromatography step (MabSelect SuRe), pseudo affinity chromatography step (Q Sepharose FF) and the virus filtration step (Planova 15N) each removed at least 3.5 logs of the model retrovirus (X-MLV) (Table4). For the virus filtration step, potential virus species are removed from the product stream based upon a size-based mechanism of separation, in which the virus is retained by the filter while rFIXFc passes through it. Importantly, the small pore size (15 nm) of the Planova 15N virus filter provides an effective barrier for a wide variety of virus types. As shown in Table4, using the Planova 15N filtration, 4.5 logs of clearance was obtained for MMV, a non-enveloped virus with an approximate diameter of 20 nm. The size differential between the relatively large enveloped viruses (≥100 nm) and 15 nm pores of the Planova filter provides an absolute barrier, with retrovirus levels below quantitation following filtration (Table4).

**Table 4:** Virus properties and virus reduction factors achieved in the rFIXFc virus clearance validation studies

			Virus reduction factor^*^				
Virus name	Virus type	Virus size (nm)	Affinity chromatography (log_10_)	Intermediate chromatography (log_10_)	Pseudo affinity chromatography (log_10_)	Planova 15N viral filtration (log_10_)	Overall reduction factor (log_10_)^†,‡^
Xenotropic murine leukaemia virus	Retrovirus (enveloped RNA virus)	80–130	4.4	2.2	3.5	>5.9^§^	>16.0^§^
Suid herpes virus 1	Enveloped DNA virus	120–200	3.7	1.0	5.3	>5.1^§^	>14.1^§^
Reovirus type 3	Non-enveloped RNA virus	60–80	3.3	0.4	6.1	>6.6^§^	>16.0^§^
Mouse minute virus	Non-enveloped DNA virus	18–22	2.9	1.2	3.3	4.5	11.9

rFIXFc, recombinant factor IX Fc fusion protein.

Numerical values shown in the table represent the reduction factor for the respective rFIXFc manufacturing process steps and viruses.

Total reduction factor (log_10_) is the sum of the lowest reduction factor value (log_10_) from duplicate determinations for each of the process steps. For the chromatography steps, the reportable value is the lowest value obtained between new and aged (cycled) chromatography resin virus clearance studies.

Reduction factor = log_10_ (viral load of input/viral load of output).

A ‘>’ indicates virus levels were below levels of quantitation for the respective steps.

The overall total virus clearance claimed for the rFIXFc purification process is >16.0 log_10_ for X-MLV, >14.1 log_10_ for SuHV-1, >16.0 log_10_ for Reo-3 and 11.9 log_10_ for MMV. These results demonstrate that the rFIXFc manufacturing process can effectively clear a broad spectrum of adventitious virus types, ranging from retroviruses to small parvoviruses.

For comparison, the virus clearance of a rFIX product produced in mammalian (CHO) cell culture is shown in Table5 8. The rFIX product manufactured in CHO cells reported virus clearance with two chromatography steps and one virus filtration step. As shown in Table5, the CHO-based rFIX process achieved >11.8 logs for Amphotropic-MLV, >14.0 logs for Herpes simplex virus, 11.3 logs for Reo-3 and 12.5 logs for Bovine parvovirus.

**Table 5:** Summary of virus properties and virus reduction factors reported for a recombinant factor IX product manufactured using a CHO cell line.^*^

			Virus reduction factor			
Virus name	Virus type	Virus size (nm)	Q Sepharose Fast Flow chromatography (log_10_)	Chelate-EMD-Cu chromatography (log_10_)	Viresolve70 viral filtration (log_10_)	Overall reduction factor (log_10_)
Amphotropic murine leukaemia virus	Retrovirus (enveloped RNA virus)	80–130	6.11	ND	>5.66	>11.8
Human herpes simplex virus	Enveloped DNA virus	150–200	4.49	3.92	>5.55	>14.0
Reovirus type 3	Non-enveloped RNA virus	60–80	5.45	0.15	5.86	11.3
Bovine parvovirus	Non-enveloped DNA virus	18–26	5.42	2.19	4.86	12.5

CHO, Chinese hamster ovary; ND, Not done.

^*^This table was adapted from *Seminars in Hematology*, 35, Adamson, Charlebois, O'Connell, and Foster, Viral Safety of Recombinant Factor IX, 22-27, Copyright Elsevier (1998) 8.

Table6 summarizes the clearance validation results for several process-related impurities, including host cell proteins, host cell DNA and recombinant protein A ligand leachate. The results demonstrated that the rFIXFc purification process provides more than four logs of clearance for protein A leachate, HEK 293H host cell proteins and HEK 293H host cell DNA. In particular, host cell DNA levels were <1 pg mg^−1^ protein, well below that considered acceptable by the WHO 19. Clearance factors were also determined for other process-related impurities, such as cell culture media additives. The results (data not shown) demonstrate sufficient degrees of removal of these impurities to ensure product safety.

**Table 6:** Summary of process-related impurity clearance for the rFIXFc manufacturing process for several process-related impurities

Process-related impurity	Impurity clearance validation scale	Overall reduction factor (log_10_)^*,†^
HEK 293H HCPs	Manufacturing	4.6
HEK 293H DNA	Manufacturing	>7.9
Recombinant protein A leachate	Laboratory	4.1

HCPs, host cell proteins; HEK, human embryonic kidney; rFIXFc, recombinant factor IX Fc.

*Overall reduction factor (log_10_) is the sum of the reduction factor values for each of the process steps validated for removal of the respective process-related impurities.

^†^Reduction factor = log_10_ (impurity load of input/impurity load of output).

## Discussion

rFIXFc is the first in a new class of long-acting clotting factors and is approved for the control and prevention of bleeding episodes, perioperative management, and routine prophylaxis in adults and children with haemophilia B (Alprolix prescribing information, Biogen Idec, Cambridge, MA). Manufacturing process validation studies demonstrated that rFIXFc possessed consistent purity, quality and safety. The validation studies also demonstrated the manufacturing process provides robust removal of potential adventitious viruses and process-related impurities. No human- or animal-derived raw materials are used during the rFIXFc cell culture and purification processes, minimizing the risk of pathogenic contamination. The process described in this study uses a well-characterized human cell line and is built upon a process developed and commonly used for the large-scale production of monoclonal antibody products 20,21. State-of-the-art chromatographic and nanofiltration technologies are used to enhance product purity and to provide robust viral clearance. These attributes collectively support a robust and reproducible manufacturing process that can support continuous supply and production of rFIXFc.

A key feature of the rFIXFc manufacturing process is the use of a human cell line (Table7). HEK 293 cells have been chosen for the production of a number of human recombinant protein therapeutics 22–26. HEK 293H cells reportedly produce FIX more efficiently than animal cell lines, with full biologic activity and greater capacity for γ-carboxylation and propeptide processing 25. The HEK293 cell line is an established expression tool that has been used extensively over the past 30 to 40 years to produce recombinant proteins for research 27; its features include amenability to transfection and high efficiency 22,23,28. An attribute of relevance to haemophilia is that the use of this cell line produces a rFIXFc molecule that displays human posttranslational modifications (PTMs) 23,27. Addition of non-human PTMs to recombinant proteins during the cell culture production process may contribute to immunogenicity 22,24,29,30, as complex recombinant proteins manufactured in non-human cell lines may bind to circulating antibodies in healthy humans 30–32. In contrast, manufacturing using human cell lines ensures that the correct translational machinery and subcellular components are present to generate a product with a human pattern of PTMs 22,27. Thus, use of a human cell line to produce recombinant proteins may protect against immunogenic epitopes, and potentially reduce the risk of developing neutralizing or non-neutralizing antidrug antibodies 22,23,26–28. In a recent study, α-Gal and N-glycolylneuraminic acid, two entities that are potentially immunogenic if present in recombinant proteins administered to humans, were not detected in rFIXFc 33–35. In contrast, characterization of several commercially available FIX and FVIII products produced in CHO or BHK cells showed detectable levels of both NGNA and α-Gal antigens 33.

**Table 7:** Comparison of rFIXFc manufacturing process with that of rFIX produced and manufactured using a CHO cell line

rFIXFc	Recombinant FIX product [29, 42, 43]
Cell line	
Human cell line (HEK 293H)	Animal cell line (CHO)
Process scale	
15 000 L Bioreactor scale	2500 L Bioreactor scale
Cell line safety	
Extensively tested and free of adventitious agents	Extensively tested and free of adventitious agents
Cell culture process	
Human/animal component free	Human/animal component free
Fed-batch culture	Batch re-feed culture
Purification process steps	
Clarification (centrifugation and depth filtration)	Two ultrafiltration steps
Three chromatography steps (protein A affinity, anion exchange and pseudo affinity chromatography)	Four chromatography steps (anion exchange [pseudo affinity], cellufine sulphate affinity, ceramic hydroxyapatite and immobilized metal ion affinity)
15 nm Planova viral filtration	Viresolve 70 virmal filtration
Ultrafiltration step	
Purification process steps validated for virus clearance	
Three chromatography steps (protein A affinity, anion exchange and pseudo affinity)	Two chromatography steps (anion exchange and immobilized metal ion affinity)
Virus filtration (Planova 15N)	Virus filtration (Viresolve 70)

CHO, Chinese hamster ovary; HEK, human embryonic kidney; rFIX, recombinant factor IX; rFIXFc, recombinant factor IX Fc.

The impurity clearance validation studies demonstrated the capacity of the manufacturing process to remove viruses of various sizes and biophysical properties, along with process-related impurities from rFIXFc, with a total reduction factor of 11.9 log_10_ or greater for each of the four model viruses evaluated. Reducing the pore size during nanofiltration has been shown to greatly enhance viral clearance without affecting the purified biologic 36. The Planova 15N filter provides robust clearance (≥4.5 logs of removal) for all four model viruses evaluated in the clearance studies.

In addition to the purification process demonstrating a high capacity for removal of adventitious viruses, the cell culture harvest for every batch is tested and confirmed to be free of adventitious viruses. In-process controls and tests are in place during cell culture and purification to ensure that they are well controlled and performing consistently, robustly and reproducibly.

The manufacturing process for rFIXFc is built upon an established, well understood manufacturing process that has been routinely and successfully applied to large-scale production of monoclonal antibodies 20,21. For preparation and production of this Fc fusion molecule, the process has been adapted to support the use of the HEK 293H cell line. Scale and continuity of production are essential features of any manufacturing process for therapeutic biologicals, and the methods described here are scaled to manufacture in a large-scale (15 000 L) production bioreactor. Furthermore, by utilizing a platform process approach, the product can be produced within any of the manufacturer's large-scale facilities, which will help to mitigate potential supply risks 37.

## Conclusion

The manufacturing process described here produces a novel, fully human, active and highly purified product free from viral contaminants or other impurities as supported by validation studies. Importantly, the manufacturing process is readily transferable and scalable to ensure consistent supply of the rFIXFc product.
